# Traumatic extracranial internal carotid-jugular fistula leading to serious injury: a case report in forensic assessment

**DOI:** 10.1080/20961790.2017.1421499

**Published:** 2018-03-16

**Authors:** Xia Liu, Lihua Fan, Chongliang Ying, Yibin Cheng, Maowen Wang

**Affiliations:** Department of Clinical Forensic Medicine, Shanghai Key Laboratory of Forensic Medicine, Shanghai Forensic Platform, Academy of Forensic Science, Shanghai, China

**Keywords:** Forensic science, carotid artery trauma, extracranial internal carotid-jugular fistula, injury degree, assessment

## Abstract

Extracranial carotid artery injuries may produce severe haemorrhage, cerebral damage or arteriovenous fistula. Examples of traumatic extracranial carotid-jugular fistula are not frequently reported, especially in forensic medicine. We report a controversial case of an extracranial internal carotid-jugular fistula resulting from a stab wound to the neck. The degree of the injury was classified under “The Standard of Human Body Injury Assessment (2014)” (SIA) in China by forensic examiners. We believe this case report will provide information for the forensic assessment of similar cases.

## Introduction

Carotid artery traumas are relatively infrequent injuries in the human body, but can have serious consequences. They have the potential to produce acute bleeding, suffocating airway compromise and debilitating cerebral damage [1–3]. A carotid-jugular fistula can be an immediate or delayed consequence of carotid artery trauma. The extracranial internal carotid artery (ICA) trauma usually follows penetrating wound of the neck, which may sometimes appear to be innocuous [4]. While, the intracranial ICA trauma is most commonly caused by a blunt injury such as from motor vehicle accident [5].

In China, the degree of the victim's injury in cases with intentional injury should be classified under the “The Standard of Human Body Injury Assessment (2014)” (SIA). Human body injuries are classified as minor injury, moderate injury or serious injury by forensic assessment that provides evidences for court trials [6]. Different depths of injury assessed by the forensic scientist may directly determine the heaviness of the sentence. According to SIA item No.5.1.2i, a traumatic cerebral infarction with signs and symptoms of neurologic impairment is classified as a serious injury, while according to SIA item No.5.1.3f, a traumatic cerebral infarction without signs and symptoms of neurologic impairment is classified as a moderate injury [7]. According to SIA item No.5.1.2n, a traumatic carotid-cavernous fistula (CCF) is a serious injury, but there are no specific items for carotid-jugular fistulas [8]. Furthermore, supplementary provision No.6.2 of the SIA stipulates that any injury that does not align with a specific item should be treated according to the item for the most similar injury. Before determining the degree of injury, the causal relationship between the attack on the victim and his injury, as well as the mechanism of injury formation should be analysed clearly.

Here, we report a case of traumatic extracranial internal carotid-jugular fistula classified as serious injury. We present the diagnosis, the mechanism of injury formation and the reasons for the forensic assessment in order to offer an insight into the similar cases.

## Case report

### The main symptoms, signs and treatment at first admission

A 45-year-old Chinese female was stabbed in the neck by a knife and was taken to hospital. Three wounds were found on her left neck, which reached the muscular layer and included bleeding. The wounds were debrided and sutured. Three days later, the victim showed decreased consciousness and was given magnetic resonance imaging (MRI) and magnetic resonance angiography (MRA) detection promptly. MRI and MRA showed scattered acute infarction (Figure S1) in the left cerebral and a hematoma around the left extracranial ICA (Figure S2). Then, she was given conservative treatment and no neurological dysfunction remained.

### The main symptoms, signs and treatment at second admission

After two months following the trauma, the victim was hospitalized again due to a vascular murmur and tremor on the left neck. One of the three scars on the left neck is near the mastoid with vascular murmur and tremor. Carotid angiography showed that a fistulous communication between the left extracranial ICA and internal jugular vein, and the left carotid artery distal to the fistula was occluded. She was treated with endovascular embolization of internal carotid-jugular fistula.

### Imaging examinations

We carefully read all the imaging data with the help of clinical radiologists. Computerized tomography (CT) scan on the victim's head did not show any obvious abnormalities in the first day following the trauma. Three days later, diffusion-weighted imaging (DWI) MRI showed multiple high signals in the left hemisphere including basal ganglia, radiation crown, centrum semiovale, frontal lobe, temporal lobe and parietal lobe, which are consistent with the imaging of acute cerebral infarction (Figure S1). Meanwhile, both MRA and computed tomography angiography (CTA) showed localized high signal intensity adjacent to the left extracranial ICA, suggesting the hematoma formation in the carotid sheath. The left intracranial ICA and left middle cerebral artery were thinner compared to the right side which accord with the left ischemic cerebral infarction (Figure S2). One month later, the left intracranial ICA was not visible by MRA, while the segmental left internal jugular vein adjacent to the hematoma was clearly visible (Figure S3). This means that the arteriovenous fistula was formed, while simultaneously, the abnormal signs associated with acute cerebral infarction were reduced. Two months later, the left intracranial ICA still was not visible, and the entire left internal jugular vein was easily identified by MRA (Figure S4).

### Forensic assessment

The victim's injury was classified as a moderate injury by the local forensic science institute according to SIA item No.5.1.3f (traumatic cerebral infarction without signs and symptoms of neurologic impairment). The victim refused to accept the result and applied for a second forensic assessment in our institute (Academy of Forensic Science). In our opinion, her injury should be classified as a serious injury based on her traumatic internal carotid-jugular fistula according to SIA item No.5.1.2n (traumatic CCF) and supplementary provision No.6.2 (any injury which does not align with a specific item should be treated according to the item for the most similar injury).

## Discussion

Both blunt and penetrating carotid artery traumas are potentially devastating, require prompt recognition and immediate repair to avoid frequent sequelae such as irreversible cerebral damage or death [2,5,9]. While, some carotid artery traumas are mild laceration, contusion or intima tearing, with only manifest pain, local swelling and slight bleeding. If the lesion of the arterial branch is not too large, the artery may go into spasm and temporarily stop bleeding. In such cases, the injuries are often asymptomatic and may be missed [10,11].

Arteriovenous fistulas can be subdivided into congenital, spontaneous and acquired [12,13]. A clear history of trauma is conducive to identify acquired arteriovenous fistula with congenital or spontaneous fistula. Acquired fistula could only have arisen in one of the two ways, i.e. from a simultaneous small injury that affected the artery and the adjacent jugular vein but was not detected at the initial examination, alternatively, raised from the hematoma and possible infection or inflammation associated with the hematoma [14,15]. Arteriovenous fistula involves abnormal traffic of blood between the artery and vein resulting in vascular murmur and tremor. However, in some cases, the symptom of arteriovenous fistula is not obvious because of the fistula's deep position or small size. Some arteriovenous fistulas without treatment may cause congestive heart failure, cerebral ischemia, thromboembolism or even rupture complications. Traumatic CCFs are almost always direct fistulas formed by a tear in the cavernous portion of ICA. The characteristic clinical features seen in patients with CCFs are the sequelae of hemodynamic dysfunction within the cavernous sinus, including proptosis, chemosis, orbital bruits and headache. This type of fistula rarely resolves spontaneously and often requires treatment. Endovascular therapy is taking the place of open surgical procedures in many cases [16,17]. There are more literatures about traumatic CCFs than those about traumatic extracranial carotid-jugular fistulas [1]. Obviously, SIA paid more attention to traumatic CCFs on account of its serious clinical sign and possible complexity of management than traumatic internal carotid-jugular fistula.

In this case, initially, the clear history of penetrating trauma on the neck, the imaging evidence of carotid artery injury and the injury's position close to the fistula suggested that the trauma can result in the arteriovenous fistula. These evidences are also important to exclude the congenital or spontaneous fistula. Furthermore, examination of the imaging showed multiple acute cerebral infarctions in the left hemisphere accorded with the regions supplied by the injured left ICA. The vasospasm of the injured artery resulted in the ipsilateral cerebral circulation disturbance [18]. Additionally, one month later, manifestations of the left ICA occlusion and carotid arteriovenous fistula formation was found by MRA and CTA [19,20]. These imaging examinations suggest that the left ICA was not completely ruptured after the trauma, and was occluded due to thrombosis over a period of time. The organized hematoma in carotid sheath then formed the wall of arteriovenous fistula. All the clinical and imaging manifestations of the victim accorded with the diagnosis of carotid-jugular fistula and the treatment is necessary. Last but not least, we classified the carotid artery injury as serious, because of not only the internal carotid-jugular fistula, but also the secondary cerebral infarction and the left ICA occlusion.

In summary, in the case of forensic assessment of a penetrating traumatic carotid-jugular fistula, the following items should be taken into account. First, it should be determined whether the wound is close to the carotid artery or not, and whether the trauma may lead to a carotid-jugular fistula. Second, the clinical and imaging manifestations should accord with the diagnosis of carotid-jugular fistula. Third, the mechanism underlying the traumatic carotid-jugular fistula can be analysed clearly. Fourth, if there is evidence such as secondary cerebral infarction, carotid artery occlusion and carotid-jugular fistula which need treatment and so on, the carotid artery trauma is proved serious. Under such circumstances, even though the fistula is not in the cavernous sinus, the injury also should be classified as a serious injury according to the special item No.5.1.2n and the supplementary provision No.6.2 of the SIA.

## Compliance with ethical standards

All procedures performed in studies involving human participants were approved by the Ethics Committee of Academy of Forensic Science, and complied with the relevant national legislation and local guidelines.

## Supplementary Material

Supp_mat_1421499_TFSR.zip
