# Supplementary figures and images for: Traumatic extracranial internal carotid-jugular fistula leading to serious injury: a case report in forensic assessment
Source: Forensic Sci Res. 2018 Mar 16;5(2):170–3. doi: 10.1080/20961790.2017.1421499 (PMC7476610; doi:10.1080/20961790.2017.1421499)

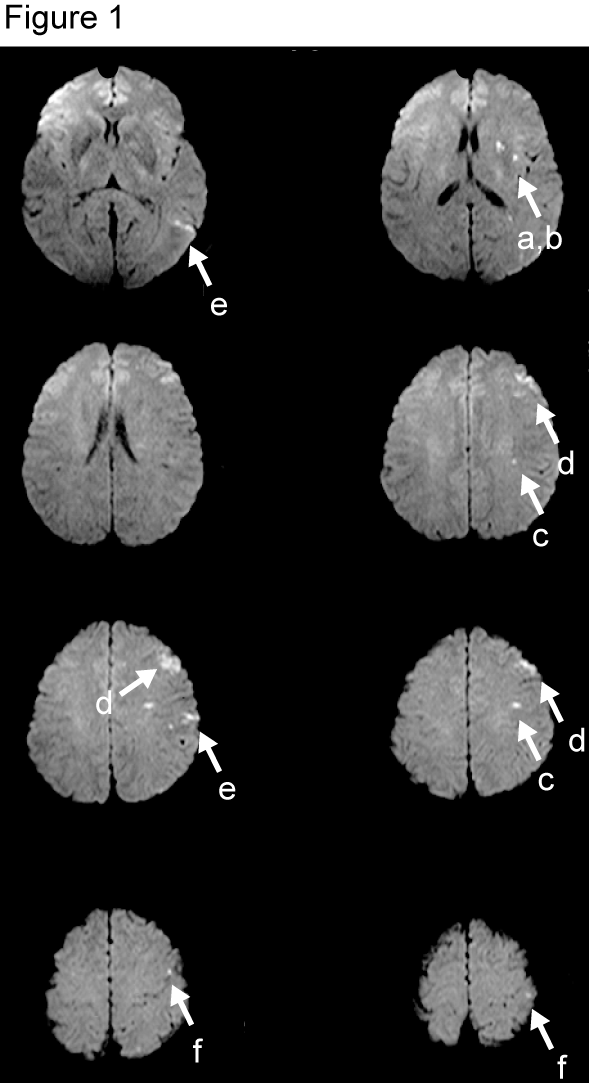

Supplement: Supp_mat_1421499_TFSR.zip [file TFSR_A_1421499_SM4173.zip › Supp_mat_1421499_TFSR/figure 1.tif]

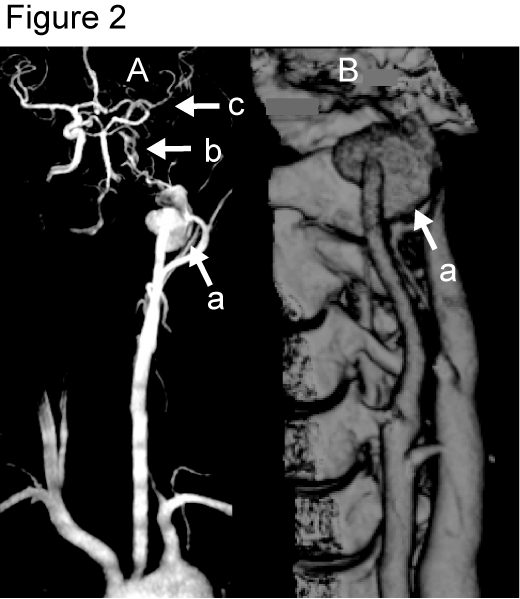

Supplement: Supp_mat_1421499_TFSR.zip [file TFSR_A_1421499_SM4173.zip › Supp_mat_1421499_TFSR/figure 2.tif]

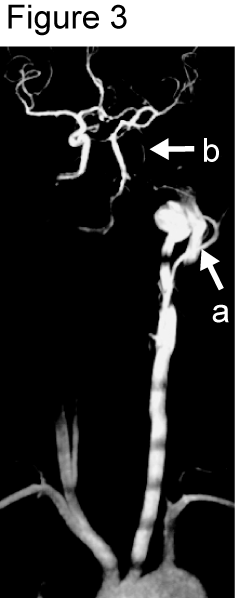

Supplement: Supp_mat_1421499_TFSR.zip [file TFSR_A_1421499_SM4173.zip › Supp_mat_1421499_TFSR/figure 3.tif]

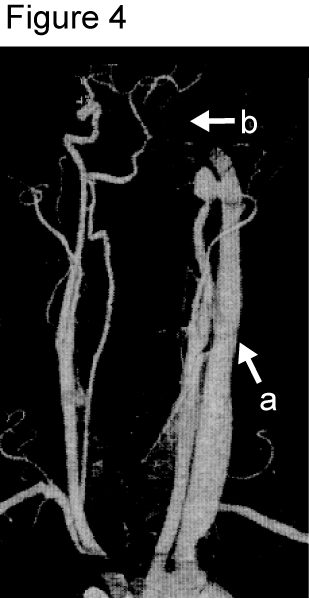

Supplement: Supp_mat_1421499_TFSR.zip [file TFSR_A_1421499_SM4173.zip › Supp_mat_1421499_TFSR/figure 4.tif]
